# Role of Dectin-2 for Host Defense against Systemic Infection with Candida glabrata

**DOI:** 10.1128/IAI.01189-13

**Published:** 2014-03

**Authors:** Daniela C. Ifrim, Judith M. Bain, Delyth M. Reid, Marije Oosting, Ineke Verschueren, Neil A. R. Gow, J. Han van Krieken, Gordon D. Brown, Bart-Jan Kullberg, Leo A. B. Joosten, Jos W. M. van der Meer, Frank Koentgen, Lars P. Erwig, Jessica Quintin, Mihai G. Netea

**Affiliations:** a Radboud University Medical Center, Department of Internal Medicine, Division of Experimental Internal Medicine, Nijmegen, The Netherlands; b Division of Applied Medicine, University of Aberdeen, Aberdeen, United Kingdom; c Aberdeen Fungal Group, Institute of Medical Sciences, University of Aberdeen, Aberdeen, United Kingdom; d Radboud University Medical Center, Department of Pathology, Nijmegen, The Netherlands; e Ozgene Pty Ltd., Bentley, Australia

## Abstract

Although Candida glabrata is an important pathogenic Candida species, relatively little is known about its innate immune recognition. Here, we explore the potential role of Dectin-2 for host defense against C. glabrata. Dectin-2-deficient (*Dectin-2*^−/−^) mice were found to be more susceptible to C. glabrata infections, showing a defective fungal clearance in kidneys but not in the liver. The increased susceptibility to infection was accompanied by lower production of T helper 1 (Th1) and Th17-derived cytokines by splenocytes of Dectin-2^−/−^ mice, while macrophage-derived cytokines were less affected. These defects were associated with a moderate yet significant decrease in phagocytosis of the fungus by the Dectin-2^−/−^ macrophages and neutrophils. Neutrophils of Dectin-2^−/−^ mice also displayed lower production of reactive oxygen species (ROS) upon challenge with opsonized C. glabrata or C. albicans. This study suggests that Dectin-2 is important in host defense against C. glabrata and provides new insights into the host defense mechanisms against this important fungal pathogen.

## INTRODUCTION

The opportunistic fungal pathogen Candida glabrata is the second most frequent cause of candidiasis after Candida albicans, accounting for approximately 15 to 25% of Candida infections (1, 2). Similar to other Candida species, C. glabrata resides as a commensal within the normal microbial flora of humans, but it may also cause serious infections in immunocompromised patients. In the last 3 decades, the number of infections due to non-albicans Candida species has increased significantly (3, 4). The current rise in the incidence of invasive C. glabrata infections is largely the result of the combinational increase in immunosuppressive infections and modern prolonged invasive medical interventions (5). Moreover, C. glabrata is of added concern due to the fact that a fraction of clinical isolates have been proven to be resistant to antifungal agents, such as azoles (6). The mortality rate associated with bloodstream infection with C. glabrata is approximately 50% in cancer patients and is even higher in bone marrow transplant patients (7, 8). However, compared to the literature on C. albicans, few studies have investigated the pathophysiology of C. glabrata infections, despite its high morbidity and mortality burden.

Pattern recognition of fungal components and activation of innate immunity are essential steps in the host defense against Candida species (9). The Candida cell wall is mainly composed of multiple layers of carbohydrates, such as mannan, β-glucan, and chitin, which are collectively recognized by C-type lectins, including macrophage mannose receptor (MR), SIGNR-1, Galectin-3, Mincle, Dectin-1, Dectin-2, and the Toll-like receptors. This recognition leads to activation of the host innate immune system. MR recognizes the *N*-linked mannans of fungal cell walls, SIGNR-1 recognizes branched α-mannans, and Galectin-3 recognizes β-(1,2)-mannans (9–11). It has recently been reported that Dectin-1 (*Clec7a*) and Dectin-2 (*Clec4n*) are specific receptors for β-glucans (12) and C. albicans-derived α-mannans (13), respectively.

Dectin-2 is a C-type lectin receptor primarily expressed by macrophages, dendritic cells and neutrophils, with specificity for structures rich in mannose (14, 15). Dectin-2 uses the FcRγ chain to signal via Syk- and caspase recruitment domain family member 9 (Card9)-dependent pathways (16–18). It represents an important receptor for the generation of the Th17-like adaptive immune response, coordinating the Th1-like responses together with Dectin-1 (17). Human Dectin-2 has a mannose recognition EPN motif (15, 19), strengthening the likelihood that Dectin-2 plays an important role in antifungal immunity. Additionally, Saijo et al. have reported that Dectin-2 is the functional receptor for α-mannans and that Dectin-2^−/−^ mice are more susceptible to C. albicans (13). However, little is known regarding the specific role of Dectin-2 for the host defense against C. glabrata.

In this study, we have explored the contribution of Dectin-2 in the pathophysiology of murine C. glabrata systemic infection. We show that the burden of C. glabrata was significantly increased in kidneys of Dectin-2^−/−^ mice, which was correlated with a decreased phagocytosis and killing of the fungus by the innate immune macrophages (as shown by live-cell video microscopy phagocytosis assays) and neutrophils (as shown by the *ex vivo* phagocytosis and killing assays). Moreover, the adaptive T helper-derived cytokine responses were also considerably affected in Dectin-2^−/−^ mice. In conclusion, our data demonstrate an important role of Dectin-2 for innate immune responses and its significant contribution to the adaptive immune responses against C. glabrata.

## MATERIALS AND METHODS

### Generation of Dectin-2^−/−^ mice.

Dectin-2^−/−^ mice were constructed by Ozgene Pty Ltd., Australia, as described previously (20). Briefly, a targeting vector was designed to introduce a conditional mutation into the mouse *Clec4n* gene (Ensembl version no. ENSMUSG00000023349; http://www.ensembl.org/Mus_musculus/Gene/Summary?db=core;g=ENSMUSG00000023349;r=6:123229843-123247021). The strategy utilizes mutant *loxP* sites (*lox66* and *lox71*) to enable an inversion of the flanked sequence in response to the expression of Cre recombinase. The recombination event inactivated the gene by switching the position and direction of the transcriptional termination (STOP) signal with *Clec4n* exon 2. The *lox66* site was inserted upstream from *Clec4n* exon 2, and a neomycin selection cassette (PGK-Neo) was targeted into the intron between exon 2 and exon 3. The selection cassette was flanked with FLP recombination target (FRT) sites to enable removal by FlpE-mediated recombination. An inverted STOP element and a *lox71* site were placed downstream from the selection marker. The 5′ and 3′ homology arms of the vector were approximately 6.1 kb and 5.9 kb, respectively. The targeting vector was linearized with PmeI and electroporated into the C57BL/6 embryonal stem (ES) cell line Bruce4. Neomycin-resistant clones were selected and screened by Southern blotting to identify homologous recombinants. Correctly targeted clones were microinjected into BALB/cJ blastocysts, which were transferred into pseudopregnant CBB6F1 foster females. Chimeric mice were obtained and outcrossed to C57BL/6J females to generate ES cell-derived targeted mutant progeny.

Eight- to 12-week-old female Dectin-2^−/−^ (*Clec4n*^−/−^) mice on a C57BL/6J background were obtained from a breeding colony at the Central Animal Laboratory, Radboud University Nijmegen Medical Centre. Age-matched C57BL/6J female mice were obtained from Charles River Wiga (Sulzfeld, Germany). All mice weighed between 20 and 25 g. The animals were fed standard laboratory chow (Hope Farms, Woerden, The Netherlands) and water *ad libitum*. Most of the experiments were repeated at least twice with a minimum of five animals per time point. All experimentation conformed to the terms and conditions of the Ethics Committee on Animal Experiments of Radboud University, Nijmegen. Key experiments, such as for fungal outgrowth and phagocytosis and killing of C. glabrata, were repeated using Dectin-2^−/−^ mice generated independently, kindly provided by Y. Iwakura (13). These validation studies were performed at the animal facility of the University of Aberdeen. Live-cell video microscopy was conducted in Aberdeen; C57BL/6 and Dectin-2^−/−^ (provided by Y. Iwakura) mice were obtained from breeding colonies. All mice were used at 8 to 16 weeks of age. Animals were kept and handled in accordance with institutional guidelines.

### Candida strains, culture media, and growth conditions.

C. glabrata CBS138 (ATCC 2001), a strain described elsewhere (21), was used in all experiments. C. glabrata was routinely grown and maintained on Sabouraud dextrose plates. For inoculum preparation, a single colony was grown in Sabouraud dextrose broth at 29°C for 24 h, with shaking. Cells were washed twice in sterile phosphate-buffered saline (PBS) and counted using a hemocytometer. Cell density was adjusted with PBS to the desired inoculum level. C. glabrata was heat killed for 30 min at 95°C. Candida albicans ATCC MYA-3573 (UC 820) was grown overnight to generate yeast cells in Sabouraud dextrose broth at 29°C, with shaking. Cells were harvested by centrifugation, washed twice with PBS, and resuspended in culture medium (RPMI 1640 Dutch modification). C. albicans yeast was heat killed for 30 min at 95°C. To generate hyphae, yeast cells were inoculated and grown overnight at 37°C in culture medium adjusted to pH 6.4 with hydrochloric acid. Hyphae were killed by exposure to 95°C for 30 min and resuspended in culture medium to a hyphal inoculum size that originated from 1 × 10^8^ CFU/ml.

### C. glabrata infection model and fungal burden.

A nonlethal experimental model of disseminated candidiasis was used, in which wild-type and Dectin-2^−/−^ mice were injected intravenously, via the lateral tail vein, with C. glabrata (1 × 10^7^ CFU/mouse) in a 100 μl volume of sterile pyrogen-free PBS. Mice were monitored daily. For survival studies, groups of 10 mice were followed-up for a period of 28 days. For immunological and histological studies, subgroups of 5 animals were killed on day 3, 7, or 14 postinfection. Tissues were collected and processed for fungal burden and cytokine analysis. To assess the tissue outgrowth of C. glabrata on these days, the liver and kidneys were removed aseptically, weighed, and homogenized in sterile PBS in a tissue grinder. The number of viable Candida cells in the tissues was determined by plating serial dilutions on Sabouraud dextrose agar plates, as described elsewhere (22). The CFU were counted after 24 h of incubation at 29°C and expressed as log CFU per gram of tissue.

### Histopathology.

Kidney samples from infected mice were kept in formalin until processed. Sections were dehydrated with xylene, rehydrated through a graded series of ethanol solutions, and stained with hematoxylin and eosin (H&E) or periodic acid-Schiff (PAS) using conventional staining methods. All individual segments were evaluated for the presence and intensity of inflammation, as well as for the presence of fungi. Tissue sections were analyzed with a VisionTek digital microscope (Sakura), using VisionTek Live software.

### *In vitro* cytokine production.

Peritoneal macrophages were isolated from mice by injecting 5 ml of ice-cold sterile PBS (pH 7.4) into the peritoneal cavity. After centrifugation and washing, cells were resuspended in RPMI 1640 culture medium containing 1 mM pyruvate, 2 mM l-glutamine, and 50 mg/liter gentamicin. Cells were counted using a Z1 Coulter particle counter (Beckman Coulter; Woerden, The Netherlands), adjusted to 1 × 10^6^ cells/ml, and cultured in 96-well round-bottom microtiter plates (Costar, Corning, The Netherlands) at 1 × 10^5^ cells/well in a final volume of 200 μl. After 24 h of incubation with different stimuli at 37°C and 5% CO_2_, the plates were centrifuged at 1,400 × *g* for 8 min, and the supernatants were collected and stored at −80°C until cytokine assays were performed.

Spleen cells were isolated by gently passing spleens through a sterile 200 μm filter chamber. After washing with sterile PBS and centrifugation at 4°C (1,200 rpm for 5 min), cells were resuspended in 2 ml RPMI 1640 in the presence of 20% fetal calf serum and counted, and concentrations were adjusted to 1 × 10^7^ cells/ml. Cells were cultured in 24-well plates (Greiner, Alphen a/d Rijn, The Netherlands) at 5 × 10^6^ cells/well, and different stimuli were added in a final volume of 1,000 μl. Supernatants were collected at two different time points (depending on the cytokine) as follows. After 48 h of incubation at 37°C and 5% CO_2_, 500 μl of supernatant per well was collected and stored at −80°C until cytokine assays were performed; thereafter, the plates were further incubated at 37°C and 5% CO_2_ for an additional 3 days. The plates were centrifuged at 1,400 × *g* for 8 min, and the remaining supernatants were collected and stored at −80°C until cytokine assays were performed. The concentrations of mouse tumor necrosis factor alpha (TNF-α), interleukin-1α (IL-1α), and IL-1β were determined by specific radioimmunoassay (RIA). Mouse IL-6, KC, IL-17, gamma interferon (IFN-γ), IL-22, and IL-10 concentrations were measured by commercial enzyme-linked immunosorbent assay (ELISA) kits (Biosource, Camarillo, CA) according to the instructions of the manufacturer.

### Phagocytosis and killing of C. glabrata.

Peritoneal macrophages and neutrophils were recruited and phagocytosis and killing assays were performed according to a modification of a method described elsewhere (23). C. glabrata/macrophage or C. glabrata/neutrophil ratios of 10:1 were used in the phagocytosis and killing studies. Peritoneal macrophages or neutrophils from groups of 5 C57BL/6J (control) and 5 Dectin-2^−/−^ mice were elicited by an intraperitoneal (i.p.) injection of heat-killed C. glabrata. Cells were collected in separate sterile tubes either 3 days (macrophages) or 4 h (neutrophils) after C. glabrata infection by washing the peritoneal cavity with 5 ml of ice-cold PBS. Phagocytes were centrifuged (for 10 min at 2,250 × *g*), counted in a Bürker counting chamber, and resuspended in RPMI 1640 Dutch modification (with 20 mM HEPES and without l-glutamine; ICN Biomedicals) supplemented with 5% heat-inactivated fetal calf serum, 1% gentamicin, 1% l-glutamine, and 1% pyruvate. The processes of phagocytosis and intracellular killing were studied in an adherent monolayer of phagocytes. To create a monolayer of phagocytes, 5 × 10^5^ cells in 100 μl of RPMI 1640 were dispensed into the wells of a 96-well flat-bottom plate (Costar) and incubated at 37°C and 5% CO_2_. Macrophages were allowed to adhere for up to 2 h, but neutrophils for only 30 min before the monolayers were gently washed with culture medium to remove nonadherent cells. The percentage of adherence was calculated as follows: (1 − [number of nonadherent cells/5 × 10^5^]) × 100. Subsequently, the cells were incubated with 1 × 10^4^ CFU C. glabrata, which were opsonized for 30 min at 24°C in modified Eagle's medium (MEM; Gibco Life Technologies) that contained 2.5% fresh mouse serum (effector/target ratio, 40:1). After 15 min, supernatants were aspirated, and monolayers were gently washed with MEM to remove noningested C. glabrata. The supernatant and well washings that contained the noningested Candida were combined and plated in serial dilutions on Sabouraud agar plates. The percentage of phagocytosed microorganisms was defined as follows: [1 − (number of uningested CFU/CFU at the start of incubation)] × 100.

The killing of C. glabrata by phagocytes was assessed in the same monolayers. After removal of the nonphagocytized Candida, 200 μl of culture medium, consisting of Sabouraud in MEM (50%, vol/vol), was added to the monolayers. After 3 h of incubation at 37°C and 5% CO_2_, the wells were gently detached with a cell scraper and washed with 200 μl distilled H_2_O to achieve lysis of phagocytes. This procedure was repeated 3 times, after which the pooled washes were adjusted to a final volume of 1 ml with distilled water. Microscopic examination of the culture plates showed that there was an almost complete removal of phagocytes. To quantify the viable intracellular Candida, 10-fold dilutions of each sample were spread on Sabouraud agar plates and incubated at 37°C for 24 h. The percentage of yeast killed by the phagocytes was determined as follows: [1 − (CFU after incubation/number of phagocytized CFU)] × 100. Phagocyte-free incubations of blastoconidia were included as a control for yeast viability.

### Live-cell video microscopy phagocytosis assays.

Standard phagocytosis assays were performed as previously described (24). Briefly, 6 × 10^5^ live C. glabrata yeasts were added to 2 × 10^5^ macrophages in μ-Slide 8-well chambers (ibidi GmbH, Germany) immediately prior to imaging. Video microscopy experiments were performed using an Ultra View VoX live-cell imaging system (PerkinElmer) with the environmental control chamber set at 37°C. Images were captured at 1-min intervals for 3 h using an electron-multiplying charge-coupled device (EMCCD) camera. At least two independent experiments were conducted for each mouse strain, and two movies for each mouse were analyzed (*n* = 7 wild-type mice, and *n* = 8 Dectin-2^−/−^ mice). Volocity 6.2.1 imaging analysis software was used to track macrophage migration at 1-min intervals for the first 60 min of the phagocytosis assay. The software enabled high-throughput analysis of macrophage migration, providing detailed information on the distances traveled and the directionality and velocity of hundreds of individual macrophages. The data were subsequently displayed in tracking diagrams and used to calculate the mean track velocity and track length of macrophages cultured with C. glabrata. The movies generated for migration analyses were also analyzed to determine phagocytic uptake at the 30-min, 60-min, and 180-min time points (25). For each mouse, 20 macrophages were analyzed for the percentage of uptake of live C. glabrata yeasts (calculated by determining the number of macrophages per 100 that had ingested at least one yeast). The phagocytic index was calculated by counting the total number of yeasts engulfed per hundred macrophages at each time point.

### Reactive oxygen species (ROS) assay.

The spontaneous and stimulus-induced oxygen radical production levels of isolated neutrophils were evaluated using luminol-enhanced chemiluminescence and determined in an automated LB96V Microlumat plus luminometer (EG & G Berthold, Bald Wilberg, Germany) as previously described (26). In this study, neutrophils (2 × 10^5^ per well) were seeded into 96-well plates and incubated in medium containing either RPMI, phorbol 12-myristate 13-acetate (PMA; 5 μg/ml), live opsonized C. albicans (10^7^ CFU/ml) or live opsonized C. glabrata (10^7^ CFU/ml). Luminol was added to each well in order to start the chemiluminescence reaction. Each measurement was carried out in at least duplicate repetitions. Chemiluminescence was determined every 145 s at 37°C for 1 h. Luminescence was expressed as relative light units (RLU) per second. Data were analyzed with Winglow software (EG & Berthold).

### Ethics statement.

All experiments in this study were carried out in strict accordance with the recommendations in the Guide for the Care and Use of Laboratory Animals of the National Institutes of Health, the Dutch law on Animal Experiments, and FELASA regulations. The protocol was approved by the Ethics Committee on Animal Experiments of the Radboud University Nijmegen Medical Centre. All efforts were made to minimize suffering of the animals. All experiments performed in Aberdeen conformed to the terms and conditions of United Kingdom Home Office licenses for research on animals and the guidelines of the University of Aberdeen ethical review committee.

### Statistical analysis.

Differences in phagocytosis, postphagocytic killing, and concentrations of cytokines were analyzed using the Mann-Whitney U test. Survival data were analyzed using the Kaplan-Meyer log rank test. Differences were considered significant at a *P* value of <0.05. The majority of the experiments was performed at least twice, and at least 5 mice/group/time point were used for the outgrowth, phagocytosis, killing, ROS, and cytokine synthesis experiments. A total of 10 mice/group were used for survival experiments. Data are presented as means ± standard errors of the means (SEM).

## RESULTS

### Increased susceptibility to C. glabrata systemic infection in Dectin-2^−/−^ mice.

**FIG 1:**
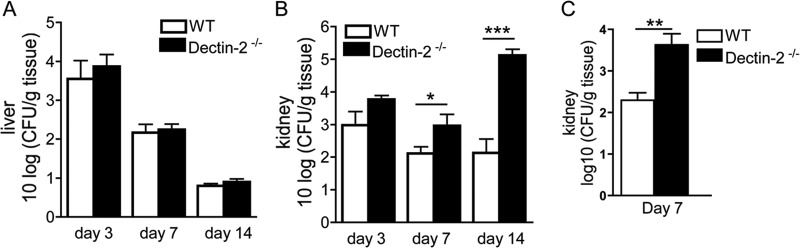
Greater C. glabrata loads in the kidneys of mice during systemic infection. (A and B) Quantification of Candida colonies from liver (A) and kidneys (B) of wild-type (WT) and Dectin-2^−/−^ mice at days 3, 7, and 14 postinfection is shown. Wild-type and Dectin-2^−/−^ mice received 10^7^ CFU of C. glabrata per mouse intravenously. After 3, 7, and 14 days, organs were collected and serial dilutions were plated on Sabouraud solid medium. Results are means ± SEM (*n* = 10 to 14 mice per group from 3 independent experiments). Significance was determined with the Mann-Whitney U test. Statistically different groups are indicated as follows: *, *P* < 0.05; ***, *P* < 0.001. (C) Fungal burdens in the kidneys of wild-type and Dectin-2^−/−^ mice provided by Y. Iwakura. Wild-type or Dectin-2^−/−^ mice received 10^7^ CFU of C. glabrata per mouse intravenously. After 7 days, kidneys were collected and serial dilutions were plated on Sabouraud agar medium. Results are means ± SEM (*n* = 12 mice per group from two independent experiments). Significance was determined with the Mann-Whitney U test. **, *P* < 0.01.

**FIG 2:**
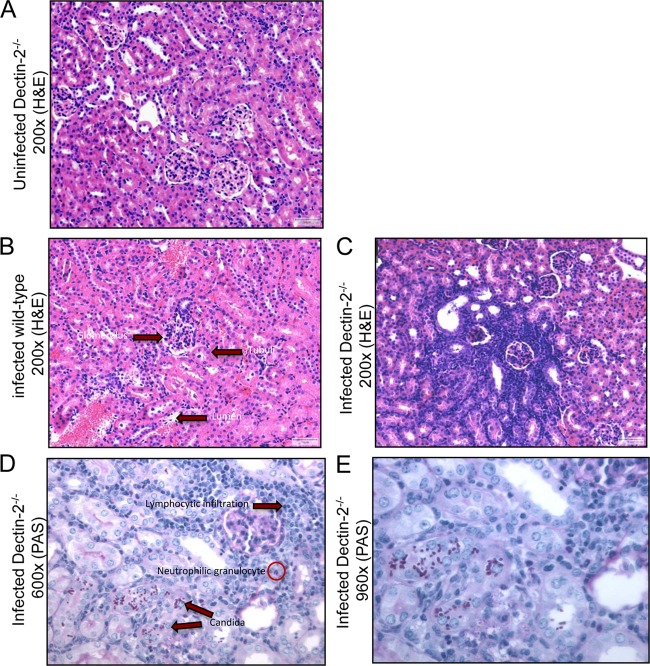
Dectin-2 deficiency affects kidney histopathology during systemic infection with C. glabrata. Histopathological assessment of kidneys in uninfected Dectin-2^−/−^ (A), infected wild-type (B), and infected Dectin-2^−/−^ (C) (hematoxylin-and-eosin [H&E]-stained sections) and infected Dectin-2^−/−^ (D and E) (periodic acid-Schiff [PAS]-stained sections) mice. Representative localized lesions and C. glabrata colonies in the kidneys of Dectin-2^−/−^ mice are shown (C, D, and E). All observations of infected kidneys were performed at day 14 after intravenous injection with C. glabrata.

To determine the role of Dectin-2 in the host defense against C. glabrata, we first compared the susceptibility to infection of Dectin-2^−/−^ and wild-type control mice. Following infection with C. glabrata, both groups of mice were able to effectively control and eradicate the fungus from the liver within 2 weeks (Fig. 1A). In contrast, while wild-type animals restrained the infection in their kidneys, significantly higher C. glabrata loads were observed in Dectin-2^−/−^ mice (Fig. 1B). Similar increases in the fungal burdens were observed in the kidneys of the Dectin-2^−/−^ mice provided by Y. Iwakura (Fig. 1C). Nonetheless, Dectin-2^−/−^ mice were less susceptible to C. glabrata than has been reported for C. albicans, with no mortality recorded in any of the mouse strains during the 28 days following intravenous infection (data not shown). Compared with the results for noninfected mice (Fig. 2A), histopathological analysis identified zones with lymphocytic infiltration suggestive of chronic inflammation in both wild-type control (Fig. 2B) and Dectin-2^−/−^ (Fig. 2C) mice. Sporadic neutrophilic granulocytes could be detected (Fig. 2D and E). At day 7 and day 14 postinfection, the kidneys of Dectin-2^−/−^ mice displayed significantly increased numbers of inflammatory foci compared with the kidneys of wild-type mice. Interestingly, fungi were only partially associated with inflammation and were often present in the lumina of tubuli, not in the tissue itself (Fig. 2D and E). Therefore, the foci of chronic, lymphocytic inflammation were not associated with the presence of fungi. In conclusion, Dectin-2 appears to be an essential pattern recognition receptor for the adequate control of C. glabrata renal infection.

### Defective Th-associated cytokine production in Dectin-2^−/−^ mice.

**FIG 3:**
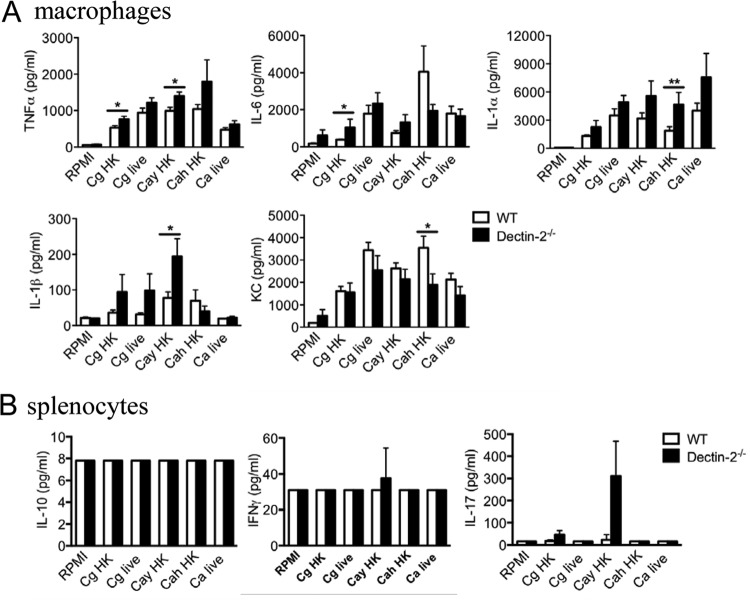
Cytokine profile from naive mice. Cells from naive mice were stimulated for 24 h (peritoneal macrophages) (A) or for 48 h and 5 days (splenocytes) (B) with heat-killed (HK) or live C. glabrata (Cg), heat-killed C. albicans yeast (Cay) or hyphae (Cah), and live C. albicans (Ca). Levels of TNF-α, IL-6, KC, IL-1α, IL-1β, IL-10, IFN-γ, and IL-17 accumulation were measured by ELISA. Results are means ± SEM (*n* = 8 mice per group). Significance was determined with the Mann-Whitney U test. Statistically different groups are indicated as follows: *, *P* < 0.05; **, *P* < 0.01.

To further characterize the role of Dectin-2 for the recognition of C. glabrata, the *ex vivo* cytokine profile was assessed in peritoneal macrophages of naive wild-type and knockout mice. Interestingly, the innate response levels from uninfected Dectin-2^−/−^ macrophages were slightly higher than the response levels of the macrophages isolated from the wild-type animals (TNF-α, IL-6, IL-1α, and IL-1β) (Fig. 3A). Only the IL-6 and KC production levels from Dectin-2^−/−^ macrophages upon stimulation with C. albicans heat-killed hyphae were lower than the levels from wild-type macrophages, although the difference reached statistical significance only for KC (Fig. 3A). Regarding the T cell-derived cytokines (IL-10, IFN-γ, and IL-17) produced by the splenocytes of uninfected mice, no differences could be detected between the two groups of mice (Fig. 3B). The IL-17 production levels in the splenocytes from Dectin-2^−/−^ mice were highly variable.

**FIG 4:**
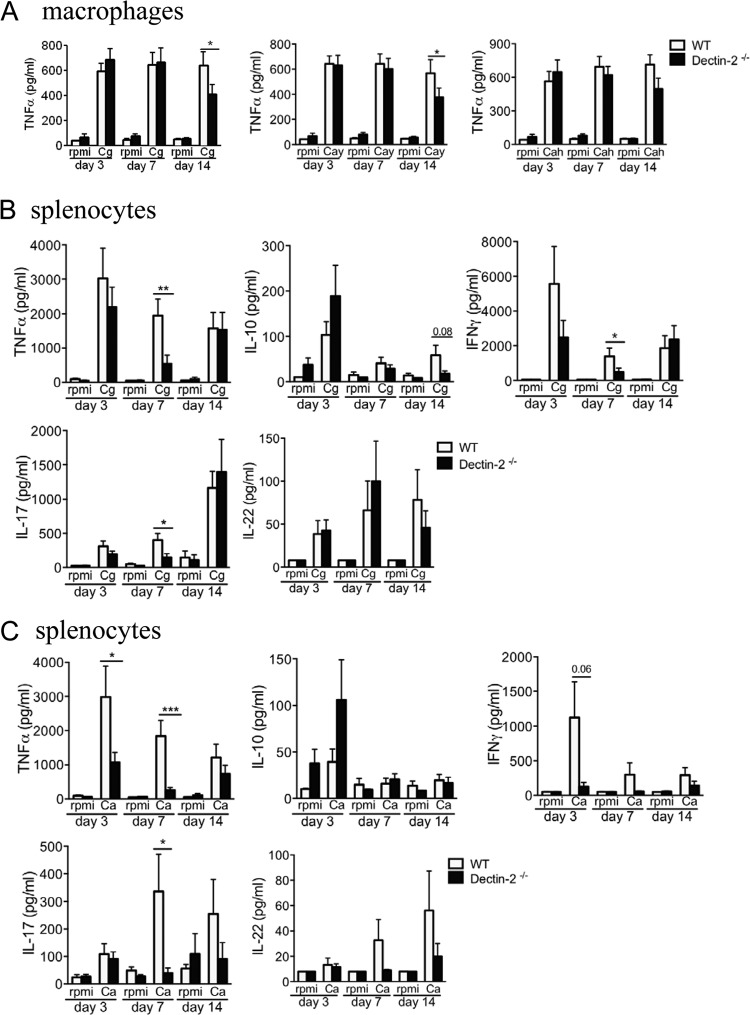
Dectin-2 is required during systemic C. glabrata infection for subsequent splenocyte recall responses. Wild-type or Dectin-2^−/−^ mice received 10^7^ CFU of C. glabrata per mouse intravenously. (A) After 3, 7, and 14 days, peritoneal macrophages were restimulated *ex vivo* with heat-killed C. glabrata (Cg), heat-killed C. albicans yeast (Cay), or hyphae (Cah) for 24 h. Levels of TNF-α accumulation in the supernatants were measured by ELISA. Results are means ± SEM (*n* = 9 or 10 mice per group) from 2 independent experiments. Significance was determined with the Mann-Whitney U test. Statistically different groups are indicated as follows: *, *P* < 0.05. (B and C) Wild-type or Dectin-2^−/−^ mice received 10^7^ CFU of C. glabrata per mouse intravenously. After 3, 7, and 14 days, splenocytes were restimulated *ex vivo* with either heat-killed C. glabrata (B) or heat-killed C. albicans yeast (C) for 48 h and 5 days. Levels of accumulation of TNF-α, IL-10, and IFN-γ (48 h) and of IL-17 and IL-22 (5 days) in the supernatants were measured by ELISA. Results are means ± SEM (*n* = 14 or 15 mice per group) from 3 independent experiments. Significance was determined with the Mann-Whitney U test. Statistically different groups are indicated as follows: *, *P* < 0.05; **, *P* < 0.01; ***, *P* < 0.001.

In a subsequent set of experiments, we studied the *ex vivo* cytokine responses of peritoneal macrophages and splenocytes isolated from mice infected with C. glabrata. No differences were detectable between the two groups of mice in most of the macrophage-derived cytokines assayed (intracellular/extracellular IL-1α and IL-1β and extracellular production of IL-6 and KC; data not shown), with the exception of TNF-α at day 14 postinfection (Fig. 4A). Similar to the results for peritoneal macrophages, *ex vivo* stimulation of splenocytes from C. glabrata-infected mice resulted in significantly lower levels of TNF-α production by Dectin-2^−/−^-deficient cells upon stimulation with either C. glabrata (Fig. 4B) or C. albicans (Fig. 4C), although dissimilarities were detectable earlier in the course of the infection. No significant differences could be detected in the amounts of the anti-inflammatory cytokine IL-10 produced by either strain. In addition to the responses of the innate cytokines, Th1 and Th17 responses at 7 days postinfection were also significantly reduced in Dectin-2^−/−^ splenocytes, as mirrored by the low production of IFN-γ and IL-17 (Fig. 4B). The levels of IL-22 were moderately reduced at day 14 postinfection (Fig. 4B). A similar pattern with more pronounced differences was observed when splenocytes from infected mice were restimulated with C. albicans yeast (Fig. 4C). As such, Dectin-2 deficiency results in decreased production of protective T helper-derived cytokines.

### Dectin-2^−/−^ peritoneal macrophages are less efficient in the uptake and killing of C. glabrata *ex vivo*.

**FIG 5:**
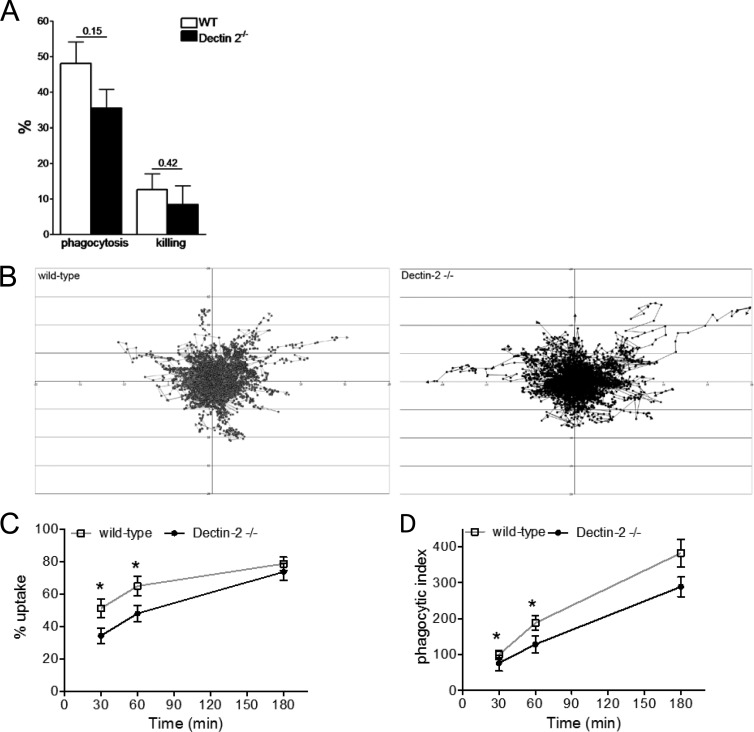
Impact of Dectin-2 deficiency on phagocytosis and killing of C. glabrata by peritoneal macrophages. (A) Phagocytosis and killing of C. glabrata cells by murine peritoneal macrophages. The results are represented as percentage of phagocytosis (the percentage of fungal cells engulfed by macrophages present in the well) and percentage of killing (the percentage of killed fungal cells among the phagocytosed yeasts). The C. glabrata/macrophage ratio was 10:1. Significance was determined with the Mann-Whitney U test. Values represent means ± SEM (*n* = 5 mice per group). (B) Migration of peritoneal macrophages was determined using live-cell video microscopy. Tracking diagrams illustrate the distances traveled and the directionality and velocity of macrophages exposed to C. glabrata at a fungal particle/macrophage ratio of 3:1. Due to the large number of macrophages tracked per video, the data were filtered to show only macrophages whose tracks lasted until 30 min to 60 min, as previous work has shown that migration activity is greater early on (25). Tracks represent the movement of individual macrophages relative to their starting position, symbols indicate the location of macrophages at 1-min intervals, and arrows represent directionality. (*n* = 7 control mice and *n* = 8 Dectin-2^−/−^ mice for 4 independent experiments). (C and D) Percentage of uptake (C) and phagocytic index (D) determined using live-cell video microscopy. The percentage of macrophages that have ingested at least one C. glabrata and the number of C. glabrata per 100 macrophages are depicted. Significance was determined with the Mann-Whitney U test. Values represent means ± SEM. *, *P* < 0.05.

The phagocytic clearance of microorganisms, including yeasts, by professional phagocytes is mediated by receptor activation that drives the engulfment and internalization of the pathogen. We therefore assessed the ability of Dectin-2^−/−^ peritoneal macrophages to recognize, phagocytose, and kill live opsonized C. glabrata. Using a classical *ex vivo* phagocytosis and killing assay (23), wild-type peritoneal macrophages were found to be slightly more efficient in the phagocytosis and subsequent killing of C. glabrata than Dectin-2^−/−^ macrophages (Fig. 5A). We further investigated the migration of peritoneal macrophages toward C. glabrata by live-cell video microscopy using a phagocytosis assay described previously (25). Dynamic analysis of individual macrophages, achieved by tracking cells to determine their directionality, distance, and velocity for the first hour of the assay, demonstrated no major differences in migration dynamics between macrophages harvested from wild-type or Dectin-2^−/−^ animals (Fig. 5B). However, in the same experiment, a complementary analysis revealed a significant difference between the two types of macrophages in terms of the uptake and phagocytosis of live C. glabrata. Following 30 min of coincubation with live C. glabrata, 51% of the wild-type macrophages had ingested at least one yeast, whereas Dectin-2^−/−^ macrophages showed a significantly reduced uptake of 34% (Fig. 5C). This significantly reduced uptake capacity was noticeable early during the phagocytosis assay (30 min and 60 min) but not at a later time point (180 min) (Fig. 5C). Additionally, the total numbers of fungal cells phagocytosed by macrophages from Dectin-2^−/−^ mice were also significantly lower than the numbers phagocytosed by wild-type macrophages at 30 min and 60 min of coincubation (Fig. 5D). Altogether, these results highlight a partial, yet significant role of Dectin-2 in mediating the uptake and engulfment of C. glabrata by macrophages. In addition, partial effects on fungal killing may also be envisioned, although more studies should be performed to establish that definitively.

### Dectin-2^−/−^ neutrophils are less efficient in phagocytosing and killing C. glabrata *ex vivo*.

**FIG 6:**
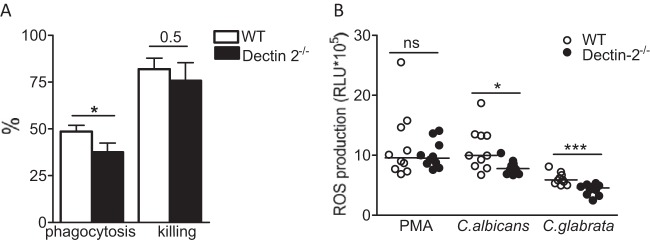
Impact of Dectin-2 deficiency on phagocytosis and killing of C. glabrata by neutrophils. (A) Phagocytosis and killing of C. glabrata cells by murine neutrophils. The results are represented as percentage of phagocytosis (the percentage of fungal cells engulfed by neutrophils present in the well) and percentage of killing (the percentage of killed fungal cells among the phagocytosed yeast). The C. glabrata/neutrophil ratio was 10:1. Values represent means ± SEM (*n* = 10 mice per group) for 2 independent experiments. *P* values are for phagocytosis or killing induced by wild-type and Dectin-2^−/−^ macrophages. *, *P* < 0.05. (B) Dectin-2^−/−^ neutrophils cannot induce the respiratory burst to the same extent as the wild type. Dectin-2^−/−^ or wild-type neutrophils were stimulated with RPMI, phorbol 12-myristate 13-acetate (PMA), live opsonized C. glabrata, or live opsonized C. albicans, and overall oxygen radical production (area under the curve after 60 min) was measured with luminol-enhanced chemiluminescence in isolated neutrophils. Data are represented as means ± SEM (*n* = 10 mice per group) for 2 independent experiments. Significance was determined with the Mann-Whitney U test. Statistically different groups are indicated as follows: *, *P* < 0.05; ***, *P* < 0.001.

We next assessed the recognition and killing of C. glabrata by neutrophils, pivotal players in the clearance of invading fungal cells from tissues. Neutrophils were harvested from the peritoneal cavity of both wild-type and Dectin-2^−/−^ mice and further subjected to an *ex vivo* phagocytosis and killing assay (23). The phagocytosis of C. glabrata by the Dectin-2^−/−^ neutrophils was considerably less effective than the phagocytosis of C. glabrata by neutrophils of wild-type mice (Fig. 6A). The killing of C. glabrata by the Dectin-2^−/−^ neutrophils was slightly diminished compared with the killing by control neutrophils (Fig. 6A). Importantly, neutrophils from Dectin-2^−/−^ mice showed a defect in secretion of ROS upon interaction with either live opsonized C. glabrata or C. albicans compared with the ROS secretion of wild-type neutrophils (Fig. 6B). Interestingly, the defect was specific for fungi, as no differences in ROS production were observed when cells were stimulated with PMA (Fig. 6B). Thus, Dectin-2 is important in both the uptake of C. glabrata by neutrophils and the subsequent ROS production and killing of the yeast.

## DISCUSSION

Dectin-2 is one of the main pattern recognition receptors for fungal mannans, with recent studies demonstrating an important role for this receptor in host defense against C. albicans (13). In the present study, we show that Dectin-2 is also an important component of the host defense against C. glabrata. The increased susceptibility of Dectin-2^−/−^ mice to a systemic infection with C. glabrata is associated with a combination of decreased production of protective T helper-derived cytokines and defective phagocytosis and killing of the yeast by both neutrophils and macrophages.

Little is known of the role of the C-type lectin receptor, Dectin-2. A recent study reported that Dectin-2^−/−^ mice are highly susceptible to a systemic infection with C. albicans (13). Moreover, it has been shown that Dectin-2 plays an important role in the induction of protective Th17 responses (17) and that, together with Dectin-3, it forms a heterodimeric pattern recognition receptor for host defense against C. albicans (27). The intracellular signaling induced by Dectin-2 involves activation of CARD9 (28), phospholipase C-γ (29), and protein kinase C-σ (30). Our study demonstrates that Dectin-2 has a nonredundant role for host defense against C. glabrata. This is an important observation not only for our improved understanding of C. glabrata infection but also for Dectin-2 biology: while Dectin-2 has been suggested to recognize mainly C. albicans hyphae (13), its importance for recognition of a Candida species that does not usually develop hyphae is a broadening of the biological importance of Dectin-2. The increased susceptibility to C. glabrata was demonstrated in two independently generated groups of Dectin-2^−/−^ mice, reinforcing this conclusion. However, these mice were less susceptible to C. glabrata than to C. albicans, as reported earlier (13). The increased susceptibility of Dectin-2^−/−^ mice to C. glabrata was apparent through their incapacity to eliminate the fungus from the kidneys, the target organ of disseminated candidiasis (31). Previously reported studies already showed that systemic candidiasis increased the fungal burden in kidneys of Dectin-1^−/−^ mice compared with the fungal burden in kidneys of the wild-type mice (12). The fact that the animals were able to eradicate C. glabrata from the liver, in comparison with the results for the kidneys, confirms recent findings concerning organ-specific Candida infection and immune responses mediated primarily by neutrophils and monocytes, as well as by various molecular factors (32–34).

Fungal pathogens have developed a number of strategies to improve their chance of survival in the host environment; e.g., C. albicans and Cryptococcus neoformans either destroy macrophages or escape via nonlytic exocytosis (35, 36). Interestingly, C. glabrata engulfed by macrophages does not undergo a morphological transition but survives and multiplies within mammalian macrophages (37, 38), which are ultimately damaged by ingested C. glabrata (38). In our study, we assessed the role of Dectin-2 for the phagocytosis and killing of C. glabrata and observed that neutrophils and macrophages lacking this receptor were less capable of ingesting and killing the yeast. Using live-cell video microscopy, we found that significantly fewer Dectin-2^−/−^ macrophages took up C. glabrata, in accordance with a lower phagocytic index where phagocytosis had occurred. These defects in the uptake and phagocytosis of C. glabrata could not be attributed to a defect in mobility. Finally, signaling through C-type lectin receptors induces cytokines and ROS that both activate the spleen tyrosine kinase (Syk)–CARD9–nuclear factor-κB (NF-κB) pathway (39) and, most likely, induce the intraphagosomal damage of fungal cells. In line with a role of the CARD9 pathway, Drewniak et al. showed that human CARD9 deficiency resulted in a selective defect in the host defense against invasive fungal infection, caused by impaired phagocyte killing in unopsonized samples compared with the phagocyte killing in opsonized samples (40). In the present study, Dectin-2^−/−^ neutrophils produced less ROS than wild-type neutrophils after stimulation of cells with either opsonized C. glabrata or C. albicans, suggesting a role of Dectin-2 in the killing of Candida species despite the presence of opsonins.

The induction of proinflammatory cytokines is an important component of anti-Candida host defense. Macrophage-derived proinflammatory cytokines like IL-1, KC, or IL-6 activate the recruitment of phagocytes and the phagocytosis and killing of fungi by phagocytes, and mice deficient in these cytokines have an increased susceptibility to systemic candidiasis (41, 42). No major defects in the production of these cytokines were observed in cells isolated from Candida-challenged Dectin-2^−/−^ mice. In contrast, the secretion of cytokines produced by Th1 and Th17 lymphocytes was significantly decreased in Dectin-2^−/−^ mice. IFN-γ is the main product of Th1 lymphocytes, and it has been proven to have a crucial role in antifungal host defense in mice (43). The defective production of IFN-γ by splenocytes of Dectin-2^−/−^ mice may represent one of the mechanisms determining the increased susceptibility to C. glabrata in these mice. Similarly, the Th17 cytokine, IL-17, represents an important component of antifungal host defense through its activation of neutrophils (44), while patients with defective Th17 responses develop chronic mucocutaneous candidiasis (45). The level of IL-17 produced by Dectin-2^−/−^ splenocytes was significantly decreased at day 7 postinfection, suggesting a role for Dectin-2 in regulating splenocyte recall responses to fungal infection. The defective Th17 responses in Dectin-2^−/−^ mice could therefore contribute to their susceptibility to C. glabrata. These results underline the conclusions of Saijo et al. (13) concerning Dectin-2-mediated immunity to C. albicans. The IL-17 defect in response to C. glabrata observed in our study was, however, less pronounced than that previously reported for C. albicans (13). Moreover, we should observe that the cytokine differences were most pronounced earlier during infection. At later time points during the infection, the picture starts to be biased by the higher fungal burden in the KO mice, which may counterbalance the defective cytokine production capacity.

The known fungal ligands recognized by Dectin-2 are α-mannans (13). Whereas significant research has been conducted on the structure of the C. albicans cell wall, much less is known regarding the cell wall components of C. glabrata. While recognition of mannans of C. glabrata by Dectin-2 is most likely, future studies should define the precise ligand-receptor interaction profile, especially as C. glabrata is less likely to form hyphae than C. albicans. Moreover, both endogenous (T cell) and exogenous (microbial carbohydrate) ligands for Dectin-2 may be important for the generation and maintenance of tolerance or the recognition of carbohydrate structures. Of particular interest is that previous reports have highlighted the ability of Dectin-2 to specifically signal after recognition of the hyphal form of C. albicans (28). The interaction between C. glabrata and Dectin-2 is different, as C. glabrata does not form traditional compartmentalized hyphae. Still, previous reports have described tubes and pseudohypha formation in several clinical isolates of C. glabrata (46), and these might be recognized by Dectin-2.

In conclusion, we demonstrate that the immune responses triggered by Dectin-2 during infection with C. glabrata might link innate immune recognition, phagocytosis, and killing of the fungus with the adaptive T helper-dependent responses. A combination of these mechanisms controls C. glabrata during systemic infection. Deciphering the precise mechanisms responsible for host defense against C. glabrata represents an important step in understanding of the pathophysiology of the disease and for the possibility to design future novel immunotherapeutic strategies.

## References

[1] Pfaller MA Messer SA Moet GJ Jones RN Castanheira M 2011 Candida bloodstream infections: comparison of species distribution and resistance to echinocandin and azole antifungal agents in intensive care unit (ICU) and non-ICU settings in the SENTRY Antimicrobial Surveillance Program (2008-2009). Int. J. Antimicrob. Agents 38:65–69. 10.1016/j.ijantimicag.2011.02.016 21514797

[2] Abi-Said D Anaissie E Uzun O Raad I Pinzcowski H Vartivarian S 1997 The epidemiology of hematogenous candidiasis caused by different Candida species. Clin. Infect. Dis. 24:1122–1128. 10.1086/513663 9195068

[3] Kauffman CA Vazquez JA Sobel JD Gallis HA McKinsey DS Karchmer AW Sugar AM Sharkey PK Wise GJ Mangi R Mosher A Lee JY Dismukes WE 2000 Prospective multicenter surveillance study of funguria in hospitalized patients. The National Institute for Allergy and Infectious Diseases (NIAID) Mycoses Study Group. Clin. Infect. Dis. 30:14–18. 10.1086/313583 10619726

[4] Ruan SY Chu CC Hsueh PR 2008 In vitro susceptibilities of invasive isolates of Candida species: rapid increase in rates of fluconazole susceptible-dose dependent Candida glabrata isolates. Antimicrob. Agents Chemother. 52:2919–2922. 10.1128/AAC.00323-08 18458136 PMC2493111

[5] Hajjeh RA Sofair AN Harrison LH Lyon GM Arthington-Skaggs BA Mirza SA Phelan M Morgan J Lee-Yang W Ciblak MA Benjamin LE Sanza LT Huie S Yeo SF Brandt ME Warnock DW 2004 Incidence of bloodstream infections due to Candida species and in vitro susceptibilities of isolates collected from 1998 to 2000 in a population-based active surveillance program. J. Clin. Microbiol. 42:1519–1527. 10.1128/JCM.42.4.1519-1527.2004 15070998 PMC387610

[6] Fidel PL Jr Vazquez JA Sobel JD 1999 Candida glabrata: review of epidemiology, pathogenesis, and clinical disease with comparison to C. albicans. Clin. Microbiol. Rev. 12:80–96 9880475 10.1128/cmr.12.1.80 PMC88907

[7] Anaissie EJ Vartivarian SE Abi-Said D Uzun O Pinczowski H Kontoyiannis DP Khoury P Papadakis K Gardner A Raad II Gilbreath J Bodey GP 1996 Fluconazole versus amphotericin B in the treatment of hematogenous candidiasis: a matched cohort study. Am. J. Med. 101:170–176. 10.1016/S0002-9343(96)80072-6 8757357

[8] Goodman JL Winston DJ Greenfield RA Chandrasekar PH Fox B Kaizer H Shadduck RK Shea TC Stiff P Friedman DJ Powderly WG Silber JL Horowitz H Lichtin A Wolff SN Mangan KF Silver SM Weisdorf D Ho WG Gilbert G Buell D 1992 A controlled trial of fluconazole to prevent fungal infections in patients undergoing bone marrow transplantation. N. Engl. J. Med. 326:845–851. 10.1056/NEJM199203263261301 1542320

[9] Netea MG Brown GD Kullberg BJ Gow NA 2008 An integrated model of the recognition of Candida albicans by the innate immune system. Nat. Rev. Microbiol. 6:67–78. 10.1038/nrmicro1815 18079743

[10] Poulain D Jouault T 2004 Candida albicans cell wall glycans, host receptors and responses: elements for a decisive crosstalk. Curr. Opin. Microbiol. 7:342–349. 10.1016/j.mib.2004.06.011 15358252

[11] Cambi A Netea MG Mora-Montes HM Gow NA Hato SV Lowman DW Kullberg BJ Torensma R Williams DL Figdor CG 2008 Dendritic cell interaction with Candida albicans critically depends on N-linked mannan. J. Biol. Chem. 283:20590–20599. 10.1074/jbc.M709334200 18482990 PMC2459306

[12] Taylor PR Tsoni SV Willment JA Dennehy KM Rosas M Findon H Haynes K Steele C Botto M Gordon S Brown GD 2007 Dectin-1 is required for beta-glucan recognition and control of fungal infection. Nat. Immunol. 8:31–38. 10.1038/ni1408 17159984 PMC1888731

[13] Saijo S Ikeda S Yamabe K Kakuta S Ishigame H Akitsu A Fujikado N Kusaka T Kubo S Chung SH Komatsu R Miura N Adachi Y Ohno N Shibuya K Yamamoto N Kawakami K Yamasaki S Saito T Akira S Iwakura Y 2010 Dectin-2 recognition of alpha-mannans and induction of Th17 cell differentiation is essential for host defense against Candida albicans. Immunity 32:681–691. 10.1016/j.immuni.2010.05.001 20493731

[14] Ariizumi K Shen GL Shikano S Ritter R III Zukas P Edelbaum D Morita A Takashima A 2000 Cloning of a second dendritic cell-associated C-type lectin (Dectin-2) and its alternatively spliced isoforms. J. Biol. Chem. 275:11957–11963. 10.1074/jbc.275.16.11957 10766825

[15] McGreal EP Rosas M Brown GD Zamze S Wong SY Gordon S Martinez-Pomares L Taylor PR 2006 The carbohydrate-recognition domain of Dectin-2 is a C-type lectin with specificity for high mannose. Glycobiology 16:422–430. 10.1093/glycob/cwj077 16423983

[16] Barrett NA Maekawa A Rahman OM Austen KF Kanaoka Y 2009 Dectin-2 recognition of house dust mite triggers cysteinyl leukotriene generation by dendritic cells. J. Immunol. 182:1119–1128 19124755 10.4049/jimmunol.182.2.1119 PMC3682801

[17] Robinson MJ Osorio F Rosas M Freitas RP Schweighoffer E Gross O Verbeek JS Ruland J Tybulewicz V Brown GD Moita LF Taylor PR Reis e Sousa C 2009 Dectin-2 is a Syk-coupled pattern recognition receptor crucial for Th17 responses to fungal infection. J. Exp. Med. 206:2037–2051. 10.1084/jem.20082818 19703985 PMC2737172

[18] Sato K Yang XL Yudate T Chung JS Wu J Luby-Phelps K Kimberly RP Underhill D Cruz PD Jr Ariizumi K 2006 Dectin-2 is a pattern recognition receptor for fungi that couples with the Fc receptor gamma chain to induce innate immune responses. J. Biol. Chem. 281:38854–38866. 10.1074/jbc.M606542200 17050534

[19] Gavino AC Chung JS Sato K Ariizumi K Cruz PD Jr 2005 Identification and expression profiling of a human C-type lectin, structurally homologous to mouse Dectin-2. Exp. Dermatol. 14:281–288. 10.1111/j.0906-6705.2005.00312.x 15810886

[20] Zhang Z Lutz B 2002 Cre recombinase-mediated inversion using lox66 and lox71: method to introduce conditional point mutations into the CREB-binding protein. Nucleic Acids Res. 30:e90. 10.1093/nar/gnf089 12202778 PMC137435

[21] Koszul R Malpertuy A Frangeul L Bouchier C Wincker P Thierry A Duthoy S Ferris S Hennequin C Dujon B 2003 The complete mitochondrial genome sequence of the pathogenic yeast Candida (Torulopsis) glabrata. FEBS Lett. 534:39–48. 10.1016/S0014-5793(02)03749-3 12527359

[22] Kullberg BJ van't Wout JW van Furth R 1990 Role of granulocytes in increased host resistance to Candida albicans induced by recombinant interleukin-1. Infect. Immun. 58:3319–3324 2144844 10.1128/iai.58.10.3319-3324.1990 PMC313656

[23] Kullberg BJ van't Wout JW Hoogstraten C van Furth R 1993 Recombinant interferon-gamma enhances resistance to acute disseminated Candida albicans infection in mice. J. Infect. Dis. 168:436–443. 10.1093/infdis/168.2.436 8335982

[24] McKenzie CG Koser U Lewis LE Bain JM Mora-Montes HM Barker RN Gow NA Erwig LP 2010 Contribution of Candida albicans cell wall components to recognition by and escape from murine macrophages. Infect. Immun. 78:1650–1658. 10.1128/IAI.00001-10 20123707 PMC2849426

[25] Lewis LE Bain JM Okai B Gow NA Erwig LP 2013 Live-cell video microscopy of fungal pathogen phagocytosis. J. Vis. Exp. 2013(71):50196. 10.3791/50196 23329139 PMC3582652

[26] Versleijen MW Oyen WJ Roelofs HM van Emst-de Vries SE Willems PH Jansen JB Wanten GJ 2008 Immune function and leukocyte sequestration under the influence of parenteral lipid emulsions in healthy humans: a placebo-controlled crossover study. Am. J. Clin. Nutr. 87:539–547 18326590 10.1093/ajcn/87.3.539

[27] Zhu LL Zhao XQ Jiang C You Y Chen XP Jiang YY Jia XM Lin X 2013 C-type lectin receptors Dectin-3 and Dectin-2 form a heterodimeric pattern-recognition receptor for host defense against fungal infection. Immunity 39:324–334. 10.1016/j.immuni.2013.05.017 23911656

[28] Bi L Gojestani S Wu W Hsu YM Zhu J Ariizumi K Lin X 2010 CARD9 mediates Dectin-2-induced IkappaBalpha kinase ubiquitination leading to activation of NF-kappaB in response to stimulation by the hyphal form of Candida albicans. J. Biol. Chem. 285:25969–25977. 10.1074/jbc.M110.131300 20538615 PMC2923990

[29] Gorjestani S Yu M Tang B Zhang D Wang D Lin X 2011 Phospholipase Cgamma2 (PLCgamma2) is key component in Dectin-2 signaling pathway, mediating anti-fungal innate immune responses. J. Biol. Chem. 286:43651–43659. 10.1074/jbc.M111.307389 22041900 PMC3243564

[30] Strasser D Neumann K Bergmann H Marakalala MJ Guler R Rojowska A Hopfner KP Brombacher F Urlaub H Baier G Brown GD Leitges M Ruland J 2012 Syk kinase-coupled C-type lectin receptors engage protein kinase C-sigma to elicit Card9 adaptor-mediated innate immunity. Immunity 36:32–42. 10.1016/j.immuni.2011.11.015 22265677 PMC3477316

[31] Spellberg B Ibrahim AS Edwards JE Jr Filler SG 2005 Mice with disseminated candidiasis die of progressive sepsis. J. Infect. Dis. 192:336–343. 10.1086/430952 15962230

[32] Lionakis MS Lim JK Lee CC Murphy PM 2011 Organ-specific innate immune responses in a mouse model of invasive candidiasis. J. Innate Immun. 3:180–199. 10.1159/000321157 21063074 PMC3072204

[33] Lionakis MS Fischer BG Lim JK Swamydas M Wan W Richard Lee CC Cohen JI Scheinberg P Gao JL Murphy PM 2012 Chemokine receptor Ccr1 drives neutrophil-mediated kidney immunopathology and mortality in invasive candidiasis. PLoS Pathog. 8:e1002865. 10.1371/journal.ppat.1002865 22916017 PMC3420964

[34] Ngo LY Kasahara S Kumasaka DK Knoblaugh SE Jhingran A Hohl TM 2014 Inflammatory monocytes mediate early and organ-specific innate defense during systemic candidiasis. J. Infect. Dis. 209:109–119. 10.1093/infdis/jit413 23922372 PMC3864383

[35] Alvarez M Casadevall A 2006 Phagosome extrusion and host-cell survival after Cryptococcus neoformans phagocytosis by macrophages. Curr. Biol. 16:2161–2165. 10.1016/j.cub.2006.09.061 17084702

[36] Bain JM Lewis LE Okai B Quinn J Gow NA Erwig LP 2012 Non-lytic expulsion/exocytosis of Candida albicans from macrophages. Fungal Genet. Biol. 49:677–678. 10.1016/j.fgb.2012.01.008 22326419 PMC3430864

[37] Kaur R Ma B Cormack BP 2007 A family of glycosylphosphatidylinositol-linked aspartyl proteases is required for virulence of Candida glabrata. Proc. Natl. Acad. Sci. U. S. A. 104:7628–7633. 10.1073/pnas.0611195104 17456602 PMC1863504

[38] Seider K Brunke S Schild L Jablonowski N Wilson D Majer O Barz D Haas A Kuchler K Schaller M Hube B 2011 The facultative intracellular pathogen Candida glabrata subverts macrophage cytokine production and phagolysosome maturation. J. Immunol. 187:3072–3086. 10.4049/jimmunol.1003730 21849684

[39] Drummond RA Saijo S Iwakura Y Brown GD 2011 The role of Syk/CARD9 coupled C-type lectins in antifungal immunity. Eur. J. Immunol. 41:276–281. 10.1002/eji.201041252 21267996 PMC3434674

[40] Drewniak A Gazendam RP Tool AT van Houdt M Jansen MH van Hamme JL van Leeuwen EM Roos D Scalais E de Beaufort C Janssen H van den Berg TK Kuijpers TW 2013 Invasive fungal infection and impaired neutrophil killing in human CARD9 deficiency. Blood 121:2385–2392. 10.1182/blood-2012-08-450551 23335372

[41] Basu S Quilici C Zhang HH Grail D Dunn AR 2008 Mice lacking both G-CSF and IL-6 are more susceptible to Candida albicans infection: critical role of neutrophils in defense against Candida albicans. Growth Factors 26:23–34. 10.1080/08977190801987513 18365876

[42] Gorjestani S Darnay BG Lin X 2012 Tumor necrosis factor receptor-associated factor 6 (TRAF6) and TGFbeta-activated kinase 1 (TAK1) play essential roles in the C-type lectin receptor signaling in response to Candida albicans infection. J. Biol. Chem. 287:44143–44150. 10.1074/jbc.M112.414276 23148225 PMC3531730

[43] Zhou P Sieve MC Bennett J Kwon-Chung KJ Tewari RP Gazzinelli RT Sher A Seder RA 1995 IL-12 prevents mortality in mice infected with Histoplasma capsulatum through induction of IFN-gamma. J. Immunol. 155:785–795 7608555

[44] Dejima T Shibata K Yamada H Hara H Iwakura Y Naito S Yoshikai Y 2011 Protective role of naturally occurring interleukin-17A-producing gammadelta T cells in the lung at the early stage of systemic candidiasis in mice. Infect. Immun. 79:4503–4510. 10.1128/IAI.05799-11 21875963 PMC3257912

[45] van de Veerdonk FL Plantinga TS Hoischen A Smeekens SP Joosten LA Gilissen C Arts P Rosentul DC Carmichael AJ Smits-van der Graaf CA Kullberg BJ van der Meer JW Lilic D Veltman JA Netea MG 2011 STAT1 mutations in autosomal dominant chronic mucocutaneous candidiasis. N. Engl. J. Med. 365:54–61. 10.1056/NEJMoa1100102 21714643

[46] Lachke SA Joly S Daniels K Soll DR 2002 Phenotypic switching and filamentation in Candida glabrata. Microbiology 148:2661–2674 12213913 10.1099/00221287-148-9-2661

